# National Compliance With Community Input on Nonprofit Hospital Community Benefit Activities

**DOI:** 10.1001/jamanetworkopen.2025.51513

**Published:** 2026-01-07

**Authors:** Ashlyn Burns, Brian Kim, Cory Cronin, Harper He, Tatiane Santos

**Affiliations:** 1 Department of Psychiatry, Indiana University School of Medicine, Indianapolis; 2 Department of Family Medicine, Indiana University School of Medicine, Indianapolis,; 3 Heritage College of Osteopathic Medicine, Ohio University, Athens; 4 College of Health Sciences and Professions, Ohio University, Athens; 5 Institute to Advance Health Equity, Ohio University, Athens; 6 Celia Scott Weatherhead School of Public Health and Tropical Medicine, Tulane University, New Orleans, Louisiana

## Abstract

**Importance:**

In exchange for tax exemptions valued at $37.4 billion, the Patient Protection and Affordable Care Act (ACA) requires all nonprofit hospitals to conduct a triennial community health needs assessment (CHNA) and develop an implementation strategy (IS). As part of these community benefit (CB) activities, hospitals must solicit and account for input from the community in the form of written comments, which may improve hospital investments in population health.

**Objective:**

To examine nonprofit hospital compliance with the community input requirement and the approaches hospitals have used to solicit and account for written comments from the community.

**Design, Setting, and Participants:**

This cross-sectional study used data from CHNAs and ISs completed by nonprofit hospitals between 2018 and 2021. CHNAs and ISs were downloaded from hospital websites in 2022. A 20% stratified random sample was drawn from all 3087 US nonprofit hospitals. Data extraction and content analysis was performed from July 2024 to March 2025, and statistical analysis was performed from March to June 2025.

**Exposure:**

ACA community input requirement in CB activities.

**Main Outcomes and Measures:**

Hospitals were considered compliant with the community input requirement if they described the following in their CHNA or IS: (1) how written comments on the most recent CHNA and/or IS were solicited, (2) at least 1 written comment received, and (3) how this information was taken into account in the current CHNA and/or IS. Bivariate analyses using χ^2^ tests to compare the organizational characteristics of compliant vs noncompliant hospitals.

**Results:**

The final study sample included 543 nonprofit hospitals, and 57 hospitals (10.5%) were compliant with the community input requirement. Among compliant hospitals, 27 (47.4%) used paper surveys, 20 (35.1%) used web-based surveys, 13 (22.8%) used in-person surveys at community outreach events, and 2 (3.5%) used telephone-based surveys to solicit community input. None of the characteristics assessed in the bivariate analysis were significantly different between compliant vs noncompliant hospitals.

**Conclusions and Relevance:**

This cross-sectional study of a nationally representative sample of nonprofit hospitals found low hospital compliance with the Internal Revenue Service community input requirement in CB documents. This finding raises questions about whether the needs and strategies identified and addressed through CB activities reflect community priorities. To ensure that CB activities address community needs as intended under the ACA, policy changes are needed to improve nonprofit hospital compliance, transparency, and accountability.

## Key Points

**Question:**

Are nonprofit hospitals complying with the federal requirement to solicit and account for written comments from the community (ie, community input requirement) on their community benefit activities, and what approaches do compliant hospitals use to solicit community input?

**Findings:**

In this nationally representative cross-sectional study of 543 hospitals, 57 (10.5%) were compliant with the Internal Revenue Service requirement to solicit and account for written comments from the community. No significant differences in hospital characteristics were found between compliant and noncompliant hospitals.

**Meaning:**

Poor hospital compliance with the community input requirement raises questions about whether community benefit activities reflect community perspectives and priorities, indicating a need for improved transparency and accountability in community benefit policies.

## Teaser

This cross-sectional study examines whether nonprofit hospitals are complying with the Internal Revenue Service’s community input requirement and assesses the approaches compliant hospitals use to solicit written comments from the community.

## Introduction

Nonprofit hospitals have been under greater scrutiny regarding their community benefit (CB) activities.^1,2,3,4,5,6,7,8,9^ In exchange for tax exemptions, nonprofit hospitals must perform CB activities to improve the well-being of their communities.^4,7,10^ In 2021, the estimated value of this tax exemption was $37.4 billion, representing the amount of money that nonprofit hospitals can reinvest in their communities.^2^ To align nonprofit hospital CB investments with community needs, since 2014, the Patient Protection and Affordable Care Act (ACA) has required all nonprofit hospitals to conduct a triennial community health needs assessment (CHNA) and develop an implementation strategy (IS).^11^ According to Internal Revenue Service (IRS) Section 501(r)(3), nonprofit hospitals are required to solicit and take into account “input from a required source representing the broad interests of the community” (ie, community input requirements).^10,11^

Compliance with the IRS community input requirement may improve hospital accountability and support more targeted allocation of CB investments.^6^ Hospitals frequently collaborate with, or at a minimum consult, public health departments when conducting their CHNA.^12^ In addition, a majority of hospitals engage community members in the process of conducting their CHNA.^6^ However, very few hospitals engage community members in the process of developing their IS.^6^ As part of IRS community input requirement, nonprofit hospitals are also required to collect “written comments received on the hospital facility’s most recently conducted CHNA and most recently adopted implementation strategy”^10^ and integrate the feedback in their CB activities. Yet, to our knowledge, no study has examined hospital compliance with this specific IRS community input requirement.

To fill this gap, we examined hospital compliance with the IRS community input requirement—specifically, the requirement to solicit written comments from community members and integrate community feedback in CB activities—using a nationally representative sample of nonprofit hospitals. We also described the approaches hospitals have used to incorporate written comments in their CB activities. In addition to illustrating the need for more transparency and accountability in CB activities, by providing example strategies that hospitals can incorporate in future CB activities, our findings may help guide noncompliant hospitals and increase the impact of CB activities on communities.

## Methods

This cross-sectional study used data extracted from CHNA and IS documents conducted by nonprofit hospitals between 2018 and 2021. A detailed description of our sampling and coding method is described elsewhere.^5^ In brief, from a total of 3087 US nonprofit hospitals we drew a 20% random sample of 613 hospitals, stratified by state to ensure representativeness. The study sample excluded 70 (11.4%) children’s hospitals, specialty hospitals, and hospitals that did not have any CHNA or IS documents. To assess the representativeness of our sample hospitals, we used bivariate analyses (ie, χ^2^ tests) to compare the characteristics of study sample hospitals to all US nonprofit hospitals. We followed the Strengthening the Reporting of Observational Studies in Epidemiology (STROBE) reporting guideline for cross-sectional studies. This study did not include human participants and used deidentified secondary data and, therefore, was exempt from institutional review board review, in accordance with 45 CFR §46.

All CHNA and IS documents were downloaded by the research team in 2022 and reviewed for data extraction using directed content analysis.^13,14^ Data extraction and content analysis was performed from July 2024 to March 2025. We were interested in determining compliance with the community input requirement, specifically the IRS requirement to solicit and take into account “written comments received on the hospital facility’s most recently conducted CHNA and most recently adopted implementation strategy” as stated in IRS Section 501(r)3.^10^ Our definition of *compliance* was based on the IRS regulatory language. That is, hospitals were considered compliant with the community input requirement if they described the following in their CHNA and/or IS: (1) how written comments on the most recent CHNA and/or IS were solicited, (2) at least 1 written comment received, and (3) how this information was taken into account in the current CHNA and/or IS. In other words, to be considered compliant, hospitals had to provide sufficient confirmation that comments were received and considered in their CHNA and/or IS documents. In conducting our content analysis, we sought to determine whether hospitals were compliant with this definition on the basis of the information reported in their CHNA and/or IS. If a hospital met all 3 criteria in either of these CB documents, this was coded as 1 (ie, compliant). If any of the criteria were not met, this was coded as 0 (ie, noncompliant).

After coding for compliance with the community input requirement, we performed a more detailed review of the CHNA and IS documents of compliant hospitals. During this second review, we extracted data on the approaches hospitals used to solicit written comments from community members. To ensure reliability, all coders initially reviewed the same set of CHNA and IS documents from 15 hospitals. We then reviewed our coding and resolved disagreements through consensus and carried the agreed-upon distinctions forward through the coding process. Upon completion of coding, we used descriptive statistics to assess the number and percentage of hospitals identified as compliant and qualitatively identified the approaches used by compliant hospitals.

### Statistical Analysis

Statistical analysis was performed from March to June 2025. We performed bivariate analyses using χ^2^ tests to compare the organizational characteristics of compliant vs noncompliant hospitals. Statistical significance was set at 2-sided *P* < .05. All statistical analyses were performed using Stata statistical software version 19 (StataCorp).

## Results

Overall, sample hospitals were comparable to all US nonprofit hospitals except that sample hospitals had a smaller percentage of hospitals that were part of a system and a larger percentage of teaching hospitals, and consisted of slightly larger hospitals (Table 1). We found that 57 of 543 hospitals in our sample (10.5%) were compliant with the community input requirement. In comparisons of compliant vs noncompliant hospitals, we did not find any statistically significant differences in organizational characteristics (Table 2).

**Table 1.** Bivariate Comparison of Sample Hospitals With All Nonprofit Hospitals in the US

Characteristic	Hospitals, No. (%)		*P* value^a^
	Total (N = 3087)	Sample (n = 543)	
Teaching	206 (6.7)	50 (9.2)	<.001
System membership	2190 (70.9)	399 (73.5)	<.001
Critical access^b^	736 (23.8)	134 (24.7)	.24
Bed size			
0-49	956 (31.0)	151 (27.8)	<.001
50-199	1155 (37.4)	186 (34.3)	
200-399	624 (20.2)	114 (21.0)	
≥400	352 (11.4)	92 (16.9)	
Area type			
Rural	532 (17.23)	101 (18.6)	.50
Micropolitan	482 (15.6)	89 (16.4)	
Metropolitan	2073 (67.2)	353 (65.0)	
Community outreach^c^	2282 (89.6)	436 (93.6)	<.001
Community health education^d^	2430 (95.37)	458 (98.3)	<.001

^a^ All bivariate analyses used χ^2^ tests.

^b^ Critical access hospital is a designation given to eligible rural hospitals that meet a list of criteria established by the Centers for Medicare & Medicaid Services (eg, ≤25 inpatient beds).

^c^ Community outreach data are based on 2548 total US nonprofit hospitals from the American Hospital Association 2018 survey; sample includes 466 hospitals.

^d^ Community health education data are based on 2548 total US nonprofit hospitals from the American Hospital Association 2018 survey; sample includes 466 hospitals.

**Table 2.** Bivariate Comparison of Hospitals That Are Compliant vs Noncompliant With Community Engagement Requirements

Characteristics	Hospitals, No. (%) (N = 543)		*P* value^a^
	Compliant (n = 57)	Noncompliant (n = 486)	
Hospitals used consulting services for CHNA^b^	27 (47.4)	221 (45.5)	.79
Hospitals collaborated with local health department in CHNA^c^	27 (47.4)	255 (52.5)	.47
Teaching	3 (5.3)	47 (9.7)	.28
System membership	40 (70.2)	359 (73.9)	.55
Critical access^d^	17 (29.8)	117 (24.1)	.34
Bed size			
0-49	16 (28.1)	135 (27.8)	.76
50-199	22 (38.6)	164 (33.7)	
200-399	9 (15.8)	105 (21.6)	
≥400	10 (17.5)	82 (16.9)	
Area type			
Rural	13 (22.8)	88 (18.1)	.69
Micropolitan	9 (15.8)	80 (16.5)	
Metropolitan	35 (61.4)	318 (65.4)	
Community outreach	49 (93.2)	387 (93.5)	.84
Community health education	52 (100)	406 (98.1)	.31

Abbreviation: CHNA, community health needs assessment.

^a^ All bivariate analyses used χ^2^ tests.

^b^ Indicates whether the hospital has used outsourced consulting services while conducting their CHNA.

^c^ Indicates whether the hospital has collaborated with local health department while conducting their CHNA.

^d^ Critical access hospital is a designation given to eligible rural hospitals that meet a list of criteria established by the Centers for Medicare & Medicaid Services (eg, ≤25 inpatient beds).

Among compliant hospitals, we identified 4 distinct approaches hospitals used to gather and incorporate community comments on the most recently completed CHNA and/or IS (Box). Paper surveys were used by 27 hospitals, representing 47.4% of compliant hospitals. Other approaches included web-based data collection (20 hospitals [35.1%]); outreach at in-person community events, such as open houses or community meetings (13 hospitals [22.8%]); and telephone-based data collection (2 hospitals [3.5%]). In most cases, compliant hospitals used more than 1 approach.

Box. **Strategies and Examples Used by 57 Hospitals to Solicit Written Comments From Community Members^a^**

**Paper Survey Responses (n = 27)**

Community surveys formatted for paper distribution
Surveys distributed to patients in waiting rooms
Surveys conducted during patient rounding

**Web-Based Data Collection (n = 20)**

Online surveys in English and Spanish
Online survey posted on hospital website
Online survey with invitation by mail
Online survey advertised in local newspaper

**Community Outreach (n = 13)**

One-on-one interviews
Questionnaires
Community forum or open house

**Telephone Data Collection (n = 2)**

Telephone surveys
Survey interviews in English and Spanish

^a^
Strategies are not exclusive (ie, hospitals may have reported the use of >1 strategy).

## Discussion

To our knowledge, this cross-sectional study is the first to evaluate nonprofit hospital compliance with the IRS community input requirement in CB documents using a nationally representative sample. Overall, we found low compliance with IRS community input requirements. Given that community engagement has been shown to improve the effectiveness and sustainability of CB activities,^15^ failing to take community input into account in CHNA and IS activities undermines the intent of the CB policy and threatens health equity.^6^ Although it is possible that hospitals may be gathering community input but not sharing this process publicly, such behavior may cloud transparency in the CB program and reflects a lack of compliance with or understanding of reporting expectations. Additional research is needed to understand levels of community input in CB activities and whether community input influences CB spending and the effectiveness of CB activities.

Among compliant hospitals, we identified 4 prevalent approaches to collect and integrate community input. The use of paper surveys was the most common approach and could be easily replicated by other hospitals. Several hospitals also reported gathering written comments from community members during in-person community outreach events, which may promote inclusion of individuals who have no access to the internet or who do not know how to use a computer. Previous research examining the strategies outlined in hospitals’ IS documents found that hospitals report plans to engage with community members through community-based events and networks such as steering committees, community boards, and coalitions.^5,16^ Hospitals can leverage these in-person community-based events to meet the IRS community input requirement, by sharing their CB documents with community members and gathering their feedback. Alternatively, hospitals can hold dedicated community meetings to share CB documents and request feedback.

Several factors may contribute to nonprofit hospital compliance with IRS requirements. Although prior research found organizational characteristics (eg, system membership, bed size, and geographic location) to be associated with the ways in which hospitals gathered community input in their IS documents,^6^ those associations were not present in the current consideration of how hospitals seek feedback from the community on completed efforts. Beyond organizational characteristics, environmental factors and regulatory oversight may also influence hospital behavior. For example, our finding of poor compliance with the community input requirement is perhaps unsurprising given poor oversight of CB activities, as detailed in a recent US Government Accountability Office report.^7^ Future research can examine other potential factors associated with hospital community engagement in CB activities, including environmental (eg, market characteristics) and regulatory (eg, state-level CB policies) factors, as well as actual CB contributions associated with the incorporation of community input.

Leveraging existing CB policies to create more transparency and accountability for nonprofit hospitals may help address low compliance with IRS community input requirements.^7^ For example, it may be beneficial to encourage hospitals to go beyond soliciting written comments and ensure that community voices are central to decision-making in CB activities. The Government Accountability Office report outlined several recommendations to improve oversight of nonprofit hospitals.^7^ For instance, a long-forgotten CB standard is related to hospital governance; specifically, nonprofit hospitals must maintain a “board of directors drawn from the community.”^7^ Nonprofit hospitals that satisfy this CB standard may improve compliance with the community input requirement and the overall quality of CB activities. At the federal level, the IRS could amend Schedule H to systematically collect information about the composition of hospitals’ boards of directors, with an emphasis on members representing the broad interests of the community. States may also implement their own CB laws to better align CB investments with the needs of local communities.

### Limitations

Our study has some limitations. Despite mandates requiring nonprofit hospitals to make their CB documents publicly available, we were not able to obtain CHNA and/or IS documents for 70 hospitals (11.4%). In addition, our definition of compliance with the community input requirement is limited to the information that hospitals describe in their CHNA and IS documents. This could lead to misclassification bias in cases where hospitals are engaging their communities and considering their feedback in CB activities, but are not including this information in their CHNA and/or IS documents.

## Conclusions

In this cross-sectional study of nonprofit hospital compliance with the IRS community input requirements, we found poor compliance with IRS community input requirements in CB documents, even though more than a decade has passed since the ACA CB requirements went into effect. The IRS community input requirement is one form of community engagement that may be a key approach to ensure alignment of CB investments with community needs. The approaches described in this study can provide pragmatic steps that hospitals can undertake to improve their compliance, CB investments, and most important, community health.

## Notes

Supplement. Data Sharing Statement
