# Supplementary material for: National Compliance With Community Input on Nonprofit Hospital Community Benefit Activities
Source: JAMA Netw Open. 2026 Jan 7;9(1):e2551513. doi: 10.1001/jamanetworkopen.2025.51513 (PMC12780923; doi:10.1001/jamanetworkopen.2025.51513)
Supplement: Supplement. — Data Sharing Statement [file jamanetwopen-e2551513-s001.pdf]

Data Sharing Statement

Burns. National Compliance With Community Input on Nonprofit Hospital Community Benefit Activities. *JAMA Netw Open*. Published January 07, 2026.
doi:10.1001/jamanetworkopen.2025.51513

Data
Data available: Yes
Data types: Data (not involving human participants)
How to access data: Data may be requested by contacting Ashlyn Burns at ashbburn@iu.edu.
When available: With publication

Supporting Documents
Document types: None

Additional Information
Who can access the data: Data will be made available to anyone requesting the data.
Types of analyses: Individuals requesting data should indicate a specified purpose for which they are requesting the data.
Mechanisms of data availability: Data will be made available with investigator support.
